# MXene supported surface plasmons on telecommunications optical fibers

**DOI:** 10.1038/s41377-022-00710-1

**Published:** 2022-01-24

**Authors:** Victor Pacheco-Peña, Toby Hallam, Noel Healy

**Affiliations:** School of Mathematics, Statistics and Physics, Newcastle University, Newcastle Upon Tyne, NE1 7RU UK

**Keywords:** Nanophotonics and plasmonics, Fibre optics and optical communications, Optical sensors, Integrated optics, Photonic devices

## Abstract

MXenes, an emerging class of two-dimensional materials, exhibit characteristics that promise significant potential for their use in next generation optoelectronic sensors. An interplay between interband transitions and boundary effects offer the potential to tune the plasma frequencies over a large spectral range from the near-infrared to the mid-infrared. This tuneability along with the ‘layered’ nature of the material not only offer the flexibility to produce plasmon resonances across a wide range of wavelengths, but also add a degree of freedom to the sensing mechanism by allowing the plasma frequency to be modulated. Here we show, numerically, that MXenes can support plasmons in the telecommunications frequency range and that surface plasmon resonances can be excited on a standard MXene coated side polished optical fiber. Thus, presenting the tantalising prospect of highly selective distributed optical fiber sensor networks.

## Abstract (web summary)

We demonstrate that MXenes can support surface plasmons at telecommunications wavelengths (1550nm) and show that surface plasmon polariton resonances can be excited on MXene coated single mode optical fibers.

## Graphical abstract

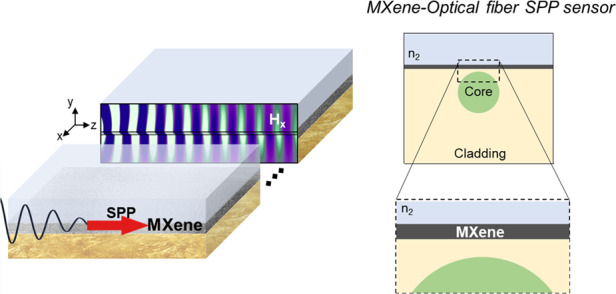

## Introduction

Two dimensional (2D) materials are a class of nanomaterials that exhibit unique physical properties due to quantum confinement effects associated with their 2D nature. Their combined superlative electronic, mechanical, and optical properties have been the driving force behind the ongoing and intense study into their applicability for opto/electronic, sensing, and energy-related applications^1–3^.

An emerging subclass of 2D materials is MXenes with a general formula M_n+1_X_n_T_x_, where *n* = 1, 2, or 3; M refers to an early transition metal element; X represents carbon or nitrogen; and T corresponds to the surface termination such as –O, –F, and –OH. MXenes are, in fact, the largest subclass of 2D materials, their physical properties are highly tuneable due to their variable chemistry^4^.

Unlike other 2D materials which mainly rely on mechanical exfoliation or bottom-up methods such as chemical vapor deposition (CVD), MXenes are mostly synthesized by selective etching of a host material, followed by liquid-based exfoliation into nanosheets^5^. MXenes nanosheet are hydrophilic and can easily form stable colloidal dispersions^6^. These properties make MXenes easily adaptable to liquid phase processing and therefore they are of great relevance for printed electronics. In fact, MXenes for printed electronics have been recently studied intensely due to the fact that their excellent solubility in aqueous solutions makes them far more attractive than other 2D materials in the context of environmentally friendly printing processes^7^.

MXenes can provide up to 95% transmittance across the visible and ultraviolet regions. Due to the free electrons of the transition metal carbide/nitride backbone, MXenes have metallic conductivity with sheet resistance shown to be better than 10 Ω☐^-1^, making them more conductive than graphene^8^. With excellent mechanical properties and tuneable optical properties, MXenes can be used as transparent conductive electrodes for applications in touch screens, flexible displays, light emitting diodes, and range of sensors^9^. For optoelectronics, MXenes have demonstrated applications in non-linear optics^10,11^, supercapacitors^8^, and biosensors^12^.

Compared to the prototypical 2D materials, MXenes can exhibit better electromagnetic properties making them a viable alternative for next generation optoelectronics applications^13^. Their metallic properties compare favourably to typical conductors such as Drude metals and low-dimensional semi-metals, making them extremely attractive for plasmonic and optical metamaterials. Furthermore, it has been shown that the plasmon energy is determined by an interplay between interband transitions and boundary effects offering the potential to directly tune plasma frequencies over a large spectral range from the near-infrared to the mid-infrared (MidIR)^14,15^. In fact, there is currently a plasmon gap within the UV-MidIR spectral window in the near infrared. In this context, the transition-metal dichalcogenides have the UV-visible range covered and Graphene and its doped counterparts span the MidIR^16,17^. Hence, the potential to support surface plasmons in the infrared offers the tantalising possibility of using MXenes for plasmonics at telecommunications wavelengths. The telecommunications regime is not only attractive as it is the wavelength window on which the internet is built, but it also allows novel devices to leverage the huge amount of investment in this operation band for alternative technologies such as sensing. Indeed, using telecoms wavelengths permits highly distributed sensing deployment as at these wavelengths the fiber is the most transparent man-made material, where photons can propagate kilometers without being lost. Furthermore, Erbium-Doped Fiber Amplifier (EDFA) amplifiers that were developed for global telecommunications networks can be employed^18^. One of the great spin-off technologies from the telecommunications platform is the optical fiber sensor, such as the fiber Bragg grating and the surface plasmon resonance fiber optic sensor^19,20^. Optical fiber sensors can be inexpensive, extremely sensitive, can be unaffected by radio frequency interference, can be positioned in hard-to-reach or view places, can be focused to measure small or precise locations, do not or will not carry electrical current (ideal for explosive hazard locations), and can be distributed over exceptionally large distances for ‘long-haul’ remote sensing. As a material for surface plasmon polariton based sensors, the porous nature of MXenes make them a particularly attractive candidate for atmospheric and gas sensing applications^21,22^. Furthermore, the large surface area of the material can be functionalized to make them hydrophilic and ready to bond to various target species, thus, furthering their potential for highly selective sensing applications.

Inspired by the interesting opportunities that MXenes can offer in different technologies, in this work we explore the possibility of using MXenes to support resonant surface plasmons polaritons (SPPs) on the side-polished optical fiber platform at telecommunications wavelengths. To provide full physical insights of the performance of such structures we first theoretically study the performance of MXenes for SPP propagation by exploiting effective medium concepts of SPPs traveling in a multi-layered media. Different thicknesses of the MXenes are considered (namely 14, 27, and 75 nm). The results are validated via numerical simulations demonstrating how SPPs can be supported in such configurations. The MXenes are then implemented in a side-polished single mode optical fiber to show its potential as a sensing device using SPPs at telecom wavelengths. The results are evaluated both numerically and theoretically (using the effective medium approach) demonstrating a good agreement between the results, corroborating the potential of the analytical model to greatly simplify the design of such devices in the future.

## Results

### Surface plasmon excitation with MXenes: analytical approach

**Fig. 1:**
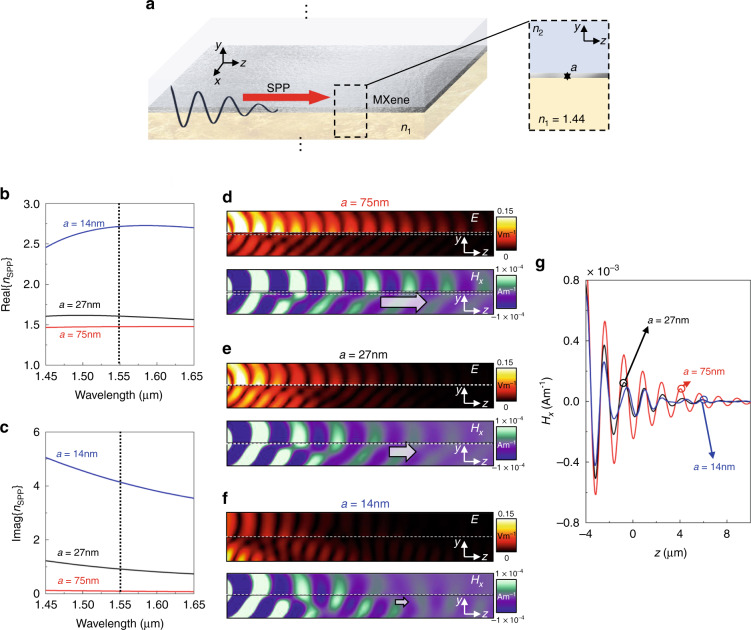
Insulator-MXene-Insulator configuration for SPP propagation. **a** Sketch of the I-MXene-I structure consisting of a Ti_3_C_2_T_x_ thin film of thickness *a* placed in between of two semi-inifnite media with refractive index *n*_1_ (SiO_2_, bottom material) and *n*_2_ (Air, top material). **b**, **c** analytical results of the real and imaginary components of the effective refractive index for the SPPs (*n*_SPP_), respectively, considering MXenes thin films with *a* = 75 nm (red), *a* = 27 nm (black) and *a* = 14 nm (blue). **d**–**f** Electric and magnetic (*H*_*x*_) field distributions on the *yz* planes showing the propagation of SPPs along the *z* axis at the telecom wavelength of λ_0_ = 1.55 µm considering MXenes with *a* = 75 nm, *a* = 27 nm, and *a* = 14 nm, respectively. **g** *H*_*x*_ field distribution along the propagation *z* axis at *y* *=* *x* *=* 0 (Air-MXene interface) for the MXenes with *a* = 75 nm (red), *a* = 27 nm (bl*a*ck) and *a* = 14 nm (blue)

To begin with, let us study the excitation of SPPs in the scenario schematically shown in Fig. 1a. In this case, we can consider a thin layer of MXene (Ti_3_C_2_T_x_) with thickness “*a*” sandwiched in between two semi-infinite dielectrics with refractive index *n*_1_ and *n*_2_ (bottom and top media, respectively). With such configuration, the excited SPPs can exist traveling along the *z* axis with the electric and magnetic fields being along the (*z,y*) and (*x*) axis, respectively (i.e., a TM wave described by ***E*** = [0, *E*_*y*_, *E*_*z*_] and ***H*** = [*H*_*x*_, 0, 0]). Based on this, the scenario depicted in Fig. 1a can be mathematically described via an effective propagation constant *β*_*SPP*_ which can be extracted by analysing the propagation of SPPs in an insulator-metal-insulator (IMI) configuration (insulator-MXene-insulator in our case). As a result, *β*_*SPP*_ can be analytically calculated by solving the following transcendental equation^23–25^:

1 $$\tanh \left( {ak_{MXene}} \right) = - \frac{{\varepsilon _1\varepsilon _{MXene}k_2k_{MXene} + \varepsilon _2\varepsilon _{MXene}k_1k_{MXene}}}{{\varepsilon _1\varepsilon _2k_{MXene}^2 + \varepsilon _{MXene}^2k_1k_2}}$$

with $k_{1,2,MXene} = \sqrt {\beta _{SPP}^2 - \varepsilon _{1,2,MXene}k_0^2}$ with subscripts 1, 2, and *MXene* representing the bottom, top, and centre media from the I-MXene-I configuration of Fig. 1a, respectively, *ε* is the complex relative permittivity of each medium and *k*_0_ is the wavenumber in free-space. Finally, we can define an effective refractive index for the SPPs propagating in such configuration as the ratio between the effective propagation constant and *k*_0_, as follows^23^:

2 $$n_{SPP} = \frac{{\beta _{SPP}}}{{k_0}}$$

Let us now evaluate the performance of the I-MXene-I structure shown in Fig. 1a for SPP excitation. Without loss of generality, we can consider that the top and bottom materials are air and silicon dioxide (SiO_2_), respectively. Let us also consider that both semi-infinite media are non-dispersive with electromagnetic parameters of *ε*_2_ = 1 (*n*_2_ = 1) and *ε*_1_ ~2.073 (*n*_1_ = 1.44), respectively. As described in the introduction, MXenes have the potential to revolutionise 2D-based technologies as they offer a range of optical, mechanical, and electronic properties, which are important in applications such as energy storage and even cancer research^4,26^. In our present work, we aim to evaluate the possibilities of using MXenes for SPP generation and show its potential use in future SPP-based technologies. As it will be discussed below, we will demonstrate how MXenes could be used, for instance, in sensing applications using optical fibers. To achieve this, here we make use of Ti_3_C_2_T_x_ thin films. The complex relative permittivity of the MXenes is modelled using an analytical fitting of the experimental data provided in ref. ^15^. We consider three thicknesses: *a* = 14 nm, *a* = 27 nm, and *a* = 75 nm (both the experimental and fitted values used in this manuscript are provided in the Supplementary Materials section 1).

With this configuration, the analytical calculations of the real and imaginary components of the effective refractive index of the SPPs (*n*_*SPP*_) calculated from Eqs. (1, 2) are shown in Fig. 1b, c, respectively, for the three values of *a* under study. Here, we evaluate the response of the I-MXene-I structure within the spectral range from 1.45–1.65 µm to show the potential of MXenes for SPP propagation at telecom wavelengths. It is important to note that these scenarios could also be implemented at longer wavelengths (potentially within the near and mid infrared) using different MXenes as long as the design wavelength is larger than the plasma wavelength, a condition needed for the existence of SPPs^23^. For the MXenes considered here, design wavelengths should be above ~1.27 µm, ~1.18 µm, and ~1.14 µm as these correspond to the plasma wavelengths for this MXene with a thickness of *a* *=* 14 nm, *a* *=* 27 nm and *a* *=* 75 nm, respectively (see Supplementary Materials Section 1 for the complex relative permittivity of the MXenes). From the results shown in Fig. 1b, c, one can observe how both the real part and imaginary components of *n*_*SPP*_ increase when reducing the thickness of the films. For instance, at the telecom wavelength of λ_0_ = 1.55 µm, Re{*n*_*SPP*_} is ~1.47, ~1.60, and ~2.71 for *a* = 75 nm, *a* = 27 nm and *a* = 14 nm, respectively while Im{*n*_*SPP*_} is ~0.09, ~0.91, and ~4.14 for the same values of *a*, respectively. In this context, it will be expected that SPPs will exist for the *a* = 75 nm MXene while they will not be strongly bounded to the interfaces for *a* = 27 nm and *a* = 14 nm within the frequency range under study due to the large losses represented by Im{*n*_*SPP*_}.

To further evaluate the response of the I-MXene-I configurations for SPPs, we carried out numerical simulations using the commercial software COMSOL Multiphysics®. Here, a 3D model was considered with SPPs in the I-MXene-I from Fig. 1a being excited via a narrow slit placed at the left-hand-side of the structure and propagating along the *z*-axis^25,27,28^. With this setup, a snapshot of the electric field and out plane (*H*_*x*_) magnetic field for the three thicknesses of the MXenes (*a* = 75 nm, *a* = 27 nm, and *a* = 14 nm) at the telecom wavelength of λ_0_ = 1.55 µm are shown in Fig. 1d, e, respectively. As observed, the SPPs are not completely clear for *a* = 27 nm and *a* = 14 nm. This is in agreement with the discussion above and with Fig. 1c where Im{*n*_*SPP*_} is very large at the telecom wavelength of 1.55 µm. However, note how the SPPs propagate in the I-MXene-I when using *a* = 75 nm (Fig. 1d). For the sake of completeness, the *H*_*x*_ field distribution on the top surface (*y* = 0) of the MXenes (Air-MXene interface) was extracted from Fig. 1d–f and the numerical results are shown in Fig. 1g, demonstrating how SPPs propagate along the *z* axis with less losses when *a* = 75 nm, in good agreement with the analytical calculations from Fig. 1b, c.

### SPP propagation in an I-MXene-I: changing the top dielectric

To fully analyse the I-MXene-I structure shown in Fig. 1a for SPP propagation, here we study the case when the top semi-infinite material is different than air. This configuration is of importance to achieve a full manipulation of SPPs given that the *n*_SPP_ can be tuned depending on the materials present in the system (as shown in Eqs. (1, 2)). This performance is known in the scientific community and has been exploited in the past in applications such as SPP-based waveguiding, sensing, focusing, and steering of SPPs^23,27,29–32^. In this context, let us focus on the MXene with *a* = 75 nm (results for values of *a* = 27 nm and *a* = 14 nm are provided in the Supplementary materials document). The bottom semi-infinite region is again SiO_2_ as in Fig. 1 while two different values are considered for the top medium (*n*_2_ = 1.25 and *n*_2_ = 1.55).

**Fig. 2:**
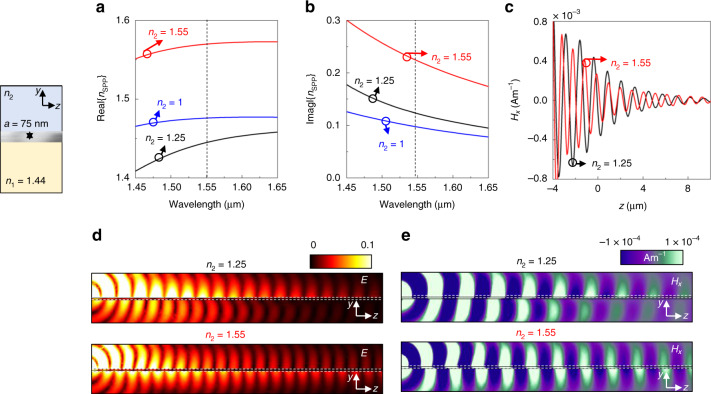
Effect of changing the top dielectric in an I-MXene-I configuration. **a**, **b** analytical results of the real and imaginary components of *n*_SPP_, respectively, considering a MXene thin film with *a* = 75 nm sandwiched in between of a semi-infinite SiO_2_ medium (bottom medium) and a dielectric medium with varying *n*_2_ (top medium), see inset on the left for the schematic representation. **c** *H*_*x*_ field distribution along the propagation *z* axis at *y* *=* *x* *=* 0 (I-MXene interface) considering an MXene with thickness *a* = 75 nm and different materials for the top semi-infinite medium: *n*_2_ = 1.25 (black) and *n*_2_ = 1.55 (red). **d**, **e** numerical results of the electric field and *H*_*x*_ field distributions on the *yz* planes, respectively, considering different materials for the top dielectric: *n*_2_ = 1._2_5 (top) and *n*_2_ = 1.55 (bottom)

With this setup, the analytical calculations of the real and imaginary components of the effective *n*_SPP_ are shown in Fig. 2a, b, respectively, considering *n*_2_ = 1.25 (black curve) and *n*_2_ = 1.55 (red curve). From these results it is clear how the effective *n*_SPP_ can be tuned by simply modifying the material on top of the MXenes. Moreover, note how the values of Im{n_SPP_} are increased when increasing *n*_2_, as expected. However, Im{n_SPP_} remain small compared to the cases shown in Fig. 1c for thinner MXenes (see also Supplementary materials for the effective *n*_SPP_ values considering MXenes with *a* = 27 nm and *a* = 14 nm with varying *n*_*2*_). For the sake of completeness, the numerical results of the *H*_*x*_ along the *z* axis at *x* *=* *y* *=* 0 are for both values of *n*_2_ are shown in Fig. 2c along with the electric field and *H*_*x*_ field distributions on the *yz* planes in Fig. 2d, e, respectively, considering *n*_2_ = 1.25 (top panels) and *n*_2_ = 1.55 (bottom panels). From these results, it can be seen how SPPs are excited in the I-MXene-I configuration, demonstrating its potential for SPP-based applications at telecom wavelengths.

### Exploiting MXenes for SPP-based telecom sensing

In the previous sections we have discussed how MXenes hold potential to support SPP propagation at telecommunication wavelengths. As SPPs can be implemented in a wide range of applications, we envision that SPP-based technologies can be revolutionized by translating well-known applications in the optical range and potentially implement them at longer wavelengths, particularly at telecom wavelengths such as those studied in this work. In this section, we demonstrate the case of a SPP-based sensor using MXenes placed on top of an optical fiber as an example of the potential of such 2D materials for SPP-based technologies. Note that, as described in the previous sections, to excite SPPs in I-MXene-I configurations, the design wavelength should be larger than the plasma wavelength of the MXenes, hence, it would be possible to design SPP-based devices at longer wavelengths. However, as our aim is to exploit MXenes together with standard telecommunications optical fibers, the spectral range is then selected to be the same as the one used in Figs. 1 and 2 (1.4–1.65 µm) as it corresponds to the low attenuation C-band spectral range (a fundamental band for telecommunication technologies).

**Fig. 3:**
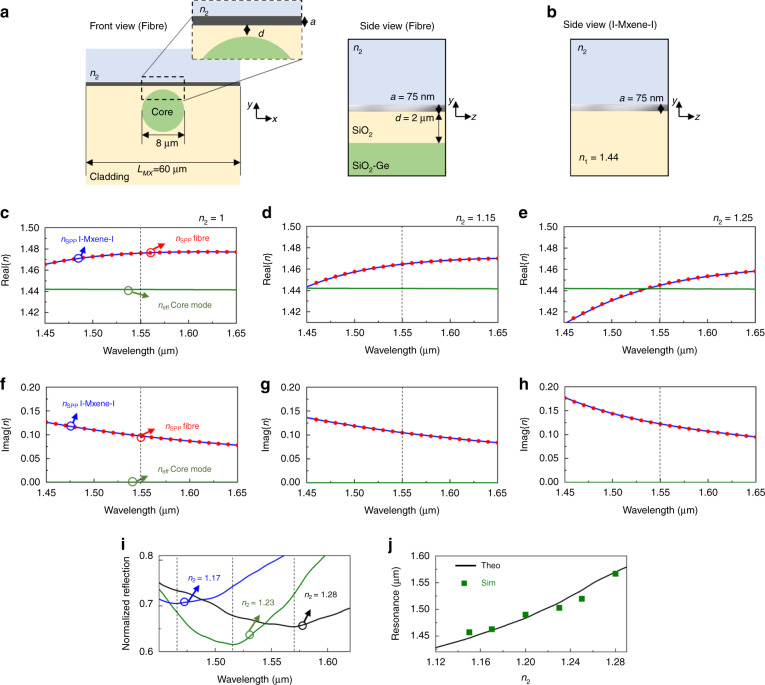
SPP-based telecom sensor: optical fiber loaded with a MXene thin film. **a** schematic representation of a single mode optical fiber loaded with a thin MXene film with height *a* = 75 nm (front and side views). The SiO_2_-Ge core has a diameter of *d*_core_ = 8 µm. **b** Sketch of the equivalent structure considered for the analytical calculations. **c**–**e** numerical results of the real part of the effective SPP mode, *n*_SPP_ (red circles) and *y* polarized core mode, *n*_eff_ (green line) along with the analytical results of the Re{n_SPP_} of an I-MXene-I structure (blue line). **f**–**h** Numerical and analytical results of the values for the imaginary component of the effective refractive index for the same modes as in (**c**–**e**). **i** Numerical results of the reflection coefficient for a 3D optical fiber sensor using loaded MXene as in (**a**) considering a top dielectric with *n*_2_ = 1.17 (blue line), *n*_2_ = 1.23 (green line) and *n*_2_ = 1.28 (black line). The spectral location of the minimum reflection are presented as black dotted lines. **j** Theoretical and numerical results of the spectral location of the resonances (minimum reflection coefficient) considering values of *n*_2_ ranging from 1.12 to 1.29

To begin with, the schematic representation of the structure under study is shown in Fig. 3a. Here we consider a single mode optical fiber consisting of a core with diameter *d*_core_ = 8 µm (made of SiO_2_ slightly doped with Germanium, Ge) having a refractive index of *n*_core_ = 1.445. The cladding is made of SiO_2_, *n*_cladd_ = 1.44. To ease the calculations, and without loss of generality, we consider the MXene to be the only dispersive media in the system. As in the previous section, here we focus our work on thin MXene films with height *a* = 75 nm having a length along the *x* axis of *L*_MX_ = 60 µm. (see Fig. 3a). Finally, the cladding on top of the core of the fiber is cut at a distance *d* from the core and the MXene is placed on top (see top inset of Fig. 3a for a schematic representation). For completeness, we provide a side view of the optical fiber sensor using MXenes on the right inset from Fig. 3a. To analytically calculate the effective refractive index of the SPP mode excited in the system shown in Fig. 3a, we can consider that such mode will be mainly bounded in the region *n*_2_-MXene-cladding. Based on this, the structure from Fig. 3a can be approximated to be equivalent to the I-MXene-I configuration discussed in Fig. 1a (and Eqs. (1 and 2)), reproduced in Fig. 3b.

With this configuration, the structure from Fig. 3a was numerically evaluated using the modal analysis from COMSOL Multiphysics® considering that the MXene is placed at a distance *d* = 2 µm from the core. Finally, the structure was numerically simulated and the real and imaginary components of the effective *n*_SPP_ were recorded for a total of 100 modes in the spectral range from 1.45 to 1.65 µm. The numerical results of the Re{*n*_SPP_}and Im{*n*_SPP_} for the SPP mode along with the effective refractive index for the *y-*polarized core mode (*n*_eff_) are shown in Fig. 3c, f, respectively, considering that the dielectric material on top of the MXene is air (*n*_2_ = 1). As observed, Re{*n*_SPP_} is different from the values of *n*_eff_ for the *y-*polarized core mode within the spectral range under study, meaning that there is no coupling between these two modes. Moreover, note that the analytical results calculated using the structure shown in Fig. 3b and defined by Eqs. (1 and 2) are also plotted as blue lines in Fig. 3c, f, demonstrating an excellent matching between them.

Now, what would happen if the top material is modified as in the I-MXene-I configuration studied in the previous section? This is an important question if one wants to exploit the structure from Fig. 3a as a sensor. To evaluate this case, the same process as in Fig. 3c, f was repeated using numerical simulations but now for values of *n*_2_ = 1.15 and *n*_2_ = 1.25. The numerical calculations for the Re{*n*_SPP_} along with the values of *n*_eff_ for the core mode are shown in Fig. 3d, e for both values of *n*_2_, respectively. The results for the imaginary components are also presented in Fig. 3g, h for completeness. As in Fig. 3c, f, the analytical values for the Re{*n*_SPP_} and Im{*n*_SPP_} were also calculated using Eqs. (1 and 2) and the results are also shown in Fig. 3d, e and Fig. 3g, h, respectively. Note that an excellent agreement is again obtained demonstrating how the analytical formulation can be accurately used to design the structure shown in Fig. 3a. By comparing the results from Fig. 3c–e, it can be seen how the Re{*n*_SPP_} is shifted towards longer wavelengths when increasing *n*_2_. Additionally, note that for *n*_2_ = 1.25, Re{*n*_SPP_} approaches to the effective refractive index of the *y-*polarized core mode (*n*_*eff*_) i.e., coupling between the two modes will exist, showing the potential for sensing applications.

**Fig. 4:**
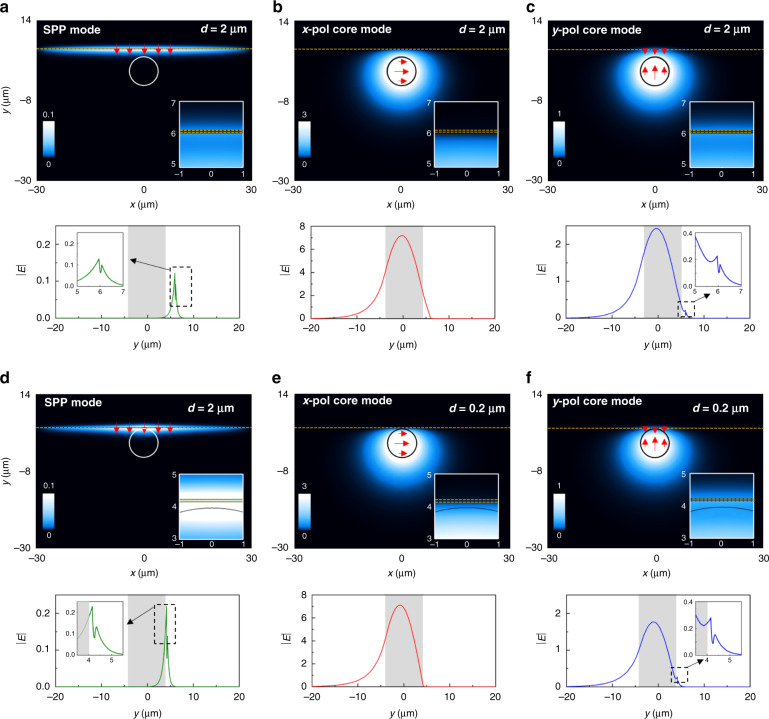
Field distributions for the SPP-fiber sensor using MXenes. **a**–**c** Numerical results of the magnitude of the electric field on the *xy* plane for the SPP, *x* and *y* polarized core modes, respectively, considering an optical fiber as in Fig. 3a when the MXene with height *a* = 75 µm is placed at a distance *d* = 2 µm from the core. The bottom panels show the electric field distribution along the y axis at *x* *=* 0. The top dielectric has a refractive index of *n*_2_ = 1.25. **d**–**f**, Same configuration as in (**a**–**c**) but with a distance *d* = 0.2 µm

To better compare the results shown in Fig. 3c–h, the numerical results of the magnitude of the electric field on the *xy* plane for the SPP mode calculated at λ_0_ = 1.55 µm using a top dielectric of *n*_2_ = 1.25 are shown in Fig. 4a along with the magnitude of the electric field along the *y* axis (at *x* = 0). As observed, the SPP mode is mainly bounded in the region *n*_2_-MXene-cladding, corroborating the assumption used for the analytical calculations, as schematically shown in Fig. 3b. Regarding the core mode, the distribution of the magnitude of the electric field for the *x* and *y* polarized modes are shown in Fig. 4b, c along with the values along the *y* axis (at *x* = 0). As observed, no coupling with the MXene is obtained for the *x* polarized core mode, given that the electric field is parallel to the thin films, meaning that this polarization will not be feasible for a sensing device. However, coupling is obtained for the *y-*polarized mode, as described before. For the sake of completeness, the numerical results of the magnitude of the electric field on the *xy* plane and along the *y* axis at *x* *=* 0 for the case with a reduced *d* *=* 0.2 µm are shown in Fig. 4d–f, demonstrating how the coupling of SPPs to the MXene is increased, as expected.

To further study the potential of MXenes for SPP-based sensing, the optical fiber from Figs. 3 and 4 was studied as a full 3D structure using the transient solver of the commercial software CST Studio Suite®, see details in the methods section. Three different materials for the top semi-infinite dielectric are considered *n*_2_ = 1.17, *n*_2_ = 1.23 and *n*_2_ = 1.28 while the rest of the parameters are the same as those from Figs. 3 and 4. The reflection coefficient was recorded for the mode shown in Fig. 4c at λ_0_ = 1.55 µm. In this context, the excitation of the SPP mode can be mapped by considering the spectral regions with small values of reflection. i.e., the *y* polarized core mode is coupled into the SPP mode. (note that the *x* polarized core mode is not used in this case as it will not couple to the MXene and hence coupling with the SPP mode will be negligible). The numerical results of the reflection coefficient within the spectral range under study for telecom sensing are shown in Fig. 3i. As it can be observed, a deep of reflection is achieved at λ_0_sim_ = 1.463 µm, 1.503 µm and 1.567 µm when *n*_2_ 1.17, *n*_2_ = 1.23 and *n*_2_ = 1.28, respectively. Note that this shifting towards longer wavelengths for larger *n*_2_ is in agreement with the discussion provided in Fig. 3c–h where the coupling of the *y* polarized core mode with the SPP mode was also red shifted.

For completeness, we carried out analytical calculations as in Fig. 3c–h considering top dielectrics with refractive index ranging from 1.12 to 1.29. The wavelength at which the effective refractive index of the *y*-polarized matches the SPP mode was recorded (Re{*n*_SPP_} = *n*_*eff*_) and the results are shown as solid black line in Fig. 3j. Moreover, the numerical results of λ_0_sim_ (deep of reflection coefficient) for different materials for the top of the MXene are shown as green squares in the same panel. From these results, one can corroborate how the spectral response is red shifted, as described before, with good agreement between the numerical and analytical values, demonstrating the potential of MXenes for SPP-based sensing at telecom wavelengths. These proof-of-concept simulations demonstrate the potential for MXene based optical fiber surface plasmon resonator devices. However, it should be noted that the large array of ‘tunable’ MXenes provide an enormous degree of freedom for device design. Our simplified effective index approach will provide a simple route to exploring this vast parameter space.

The proposed MXene-based structures for SPP-based devices at telecom wavelengths could be fabricated by the Langmuir-Blodgett (LB) technique. In this technique nanosheets can be suspended on a liquid subphase while a thinned fiber surface is passed through the liquid-air interface depositing as well-tiled monolayer. LB has been demonstrated experimentally for Ti_3_C_2_T_x_ recently by pH controlled water to prevent loss to the subphase^33^. Another study, instead, used a liquid-liquid interface with chloroform on a water subphase to drive monolayer formation^34^. Multiple LB layers can then be deposited sequentially to create MXene adlayers with the desired thickness.

## Discussion

In summary, we report that the tunable metal-like properties of MXenes can be harnessed to support the excitation and propagation of surface plasmon polaritons in the telecommunications wavelength regime. Furthermore, we show that plasmon resonances can be excited and propagated on MXene coated side-polished optical fibers, thus presenting the prospect of MXenes being an attractive plasmonic material for distributed fiber optical sensors. It is anticipated that the ‘layered’ nature of this material and its tunability will prove highly advantageous for such sensing devices. Finally, we present an analytic effective medium model that agrees very well with numerical simulations, thus providing a simplified approach to designing such devices in the future. Importantly, MXenes are the largest subgroup of 2D materials and the tunability of their optical properties mean that there are many possible device configurations. In this context, the proposed theoretical model is a much faster and computationally inexpensive method of predicting/selecting device configurations and it could be implemented in real-time sensing applications.

## Materials and methods

The numerical simulations of Figs. 1 and 2 were performed using the frequency-domain solver of the commercial software COMSOL Multiphysics^®^ by considering a 3D model of the I-MXene-I configuration. The excitation of the SPPs on the I-MXene-I configurations was applied by using a thin slit placed on the left of the structure with a width (along the *z* axis) of 0.1λ_0_ (λ_0_ = 1.55 µm)^25,27^. For the simulations from Fig. 3c–h and Fig. 4, a 2D model of the transversal cross-section of the optical fiber from Fig. 3a was modelled and studied using the mode analysis solver from COMSOL Multiphysics^®^. The numerical results for the 3D SPP-based sensor using an optical fiber loaded with MXenes (Fig. 3i, j) were carried out using the transient solver of the commercial software CST Studio Suite® with an adaptative mesh refinement. Open boundary conditions were on the bottom, top, left, right boundaries of the simulation box to avoid undesirable reflections.

## Supplementary information

Supplementary materials final version
