# Supplementary material for: MXene supported surface plasmons on telecommunications optical fibers
Source: Light Sci Appl. 2022 Jan 24;11:22. doi: 10.1038/s41377-022-00710-1 (PMC8784538; doi:10.1038/s41377-022-00710-1)
Supplement: Supplementary file 1 — Supplementary materials final version [file 41377_2022_710_MOESM1_ESM.pdf]

Supplementary materials

MXene Supported Surface Plasmons on Telecommunications Optical Fibers

Victor Pacheco-Pena[1], Toby Hallam[1] and Noel Healy[1*]*

[1]*School of Mathematics, Statistics and Physics, Newcastle University, Newcastle Upon Tyne, NE1 7RU, United Kingdom*
**email(s): victor.pacheco-pena@newcastle.ac.uk, noel.healy@newcastle.ac.uk*

1. **Complex permittivity MXenes**
2. **Complex effective refractive index SPPs. MXenes of thickness 27 nm and 14 nm**
3. **Field distributions Insulator-MXene-Insulator**

1. Complex permittivity MXenes

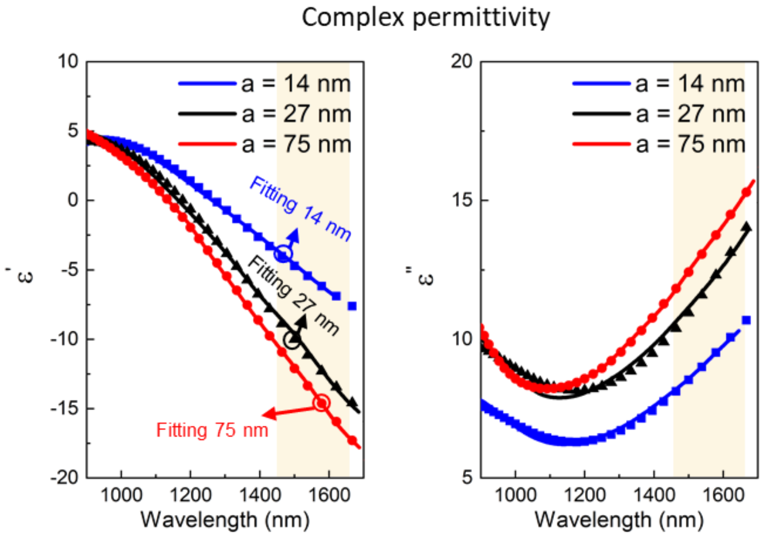

Fig. S1| Complex permittivity MXenes. Experimental (solid lines, extracted from [1]) and fitted values (symbols) of the real (left) and imaginary (right) parts of the complex permittivity for MXenes with thickness of *a* = 14 nm (blue), *a* = 27 nm (black) and *a* = 75 nm (red).

2. Complex effective refractive index SPPs. MXenes of thickness 27 nm and 14 nm

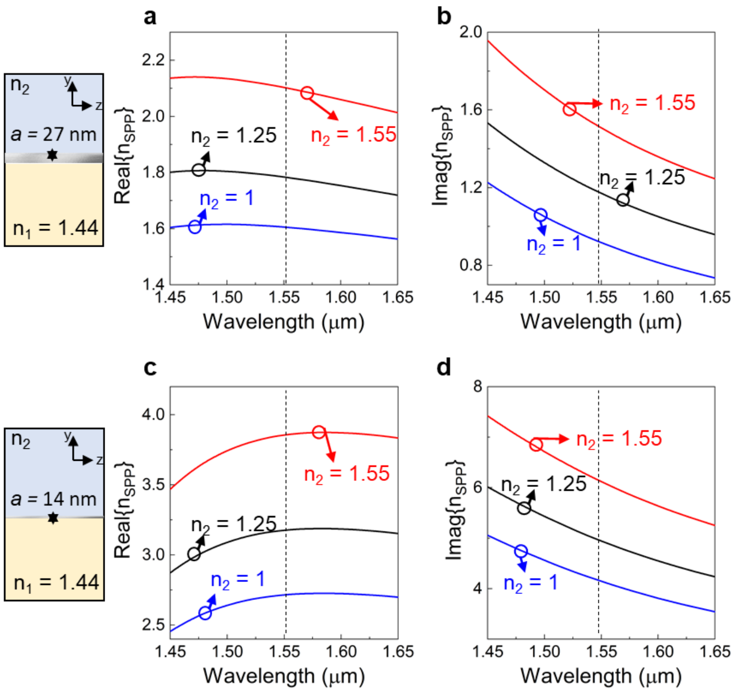

Fig. S2| Insulator-MXene-Insulator. analytical results of the **a,c** real and **b,d** imaginary components of n_{SPP}, considering a MXene thin film with *a* = 27 nm (first row) and *a* = 14 nm (second row) sandwiched in between of a semi-infinite SiO_2 medium (bottom medium) and a dielectric medium with varying n_2 (top medium), see inset on the left for the schematic representation.

3. Field distributions Insulator-MXene-Insulator

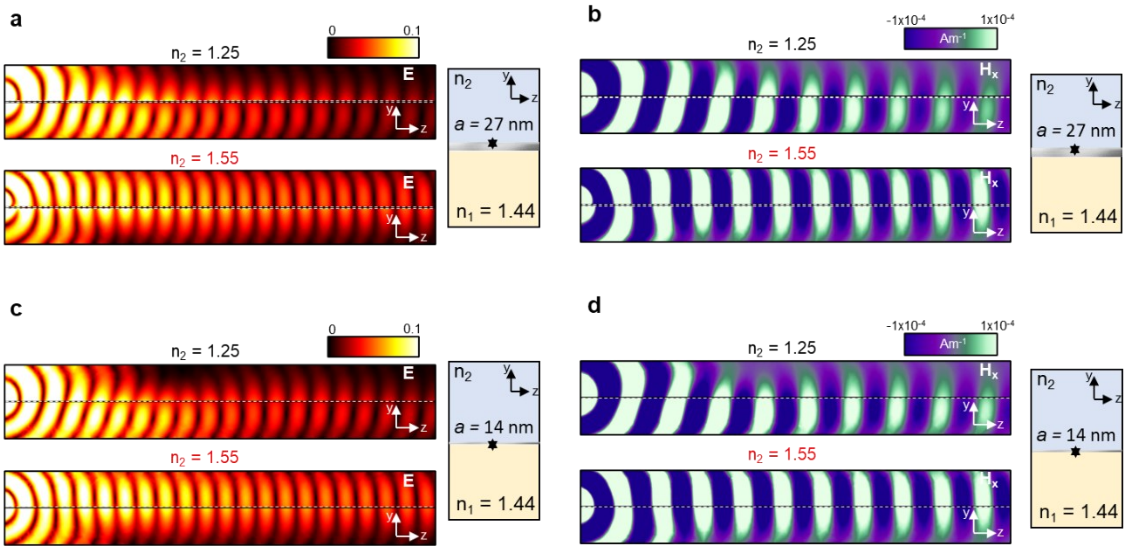

Fig. S3| Field distributions Insulator-MXene-Insulator. numerical results of the **a,c** electric field and **b,d** H_x field distributions on the *yz* planes, respectively, considering different materials for the top dielectric: $n_2 = 1.25$ (top) and $n_2 = 1.55$ (bottom) for I-MXene-I configurations using MXenes of thickness *a* = 27 nm (first row) and *a* = 14 nm (second row).

References

1. Dillon, A. D. *et al.* Highly Conductive Optical Quality Solution-Processed Films of 2D Titanium Carbide. *Adv. Funct. Mater.* **26**, 4162–4168 (2016).
